# Acute Effects of the Novel Psychoactive Drug 2C-B on Emotions

**DOI:** 10.1155/2015/643878

**Published:** 2015-10-12

**Authors:** Débora González, Marta Torrens, Magí Farré

**Affiliations:** ^1^Department of Pharmacology and Department of Psychiatry, Universitat Autònoma de Barcelona, Doctor Aiguader 80, 08003 Barcelona, Spain; ^2^Hospital del Mar Medical Research Institute (IMIM), Doctor Aiguader 88, 08003 Barcelona, Spain; ^3^Institute of Neuropsychiatry and Addictions (INAD), Parc de Salut Mar, Paseo Marítimo 25, 08003 Barcelona, Spain; ^4^Hospital Universitari Germans Trias i Pujol-HUGTP-IGTP, Carretera de Canyet, s/n, 08916 Badalona, Spain

## Abstract

*Background*. 2C-B (Nexus) is one of the most widespread novel psychoactive substances. There is limited information about its pharmacological properties, and few studies in humans concern its acute and chronic effects. 2C-B has been classified as a stimulant, hallucinogen, entactogen, and/or empathogen. *Objectives*. To evaluate the emotional, subjective, and cardiovascular effects of 2C-B. *Methods*. Twenty healthy recreational 2C-B users (12 women) self-administered a 20 mg dose of 2C-B. Evaluations included emotional (IAPS, FERT, and speech), subjective (visual analog scales, ARCI, VESSPA, HRS, and POMS questionnaires), and cardiovascular effects (blood pressure and heart rate). *Results*. Positive subjective effects predominated with a reduction of anger under the influence of 2C-B. It did, however, increase reactivity to negative emotional stimuli and decrease the ability to recognize expressions of happiness. Augmented emotionality in speech could be appreciated by others. 2C-B induced euphoria and well-being, changes in perceptions, and slight hallucinogenic states. Mild sympathetic actions were observed. *Conclusions*. The specific profile that 2C-B exerts on emotions suggests its classification as an entactogen with psychedelic properties.

## 1. Introduction

**Figure 1:**
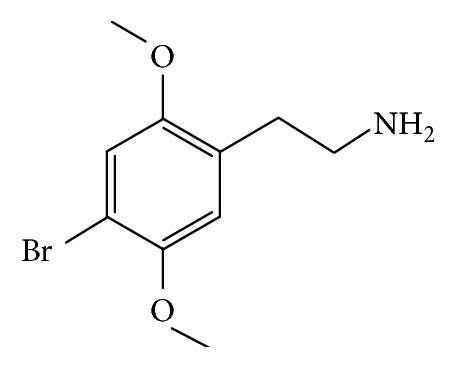
Chemical structure of 2C-B.

Alexander Shulgin synthesized 4-bromo-2,5-dimethoxyphenethylamine (2C-B, Nexus, Figure 1), a phenylethylamine with hallucinogenic effects, in 1974 [1]. Nowadays, 2C-B is one of the most widespread novel psychoactive substances (NPS) in Europe [2] and Australia [3]. It is, moreover, commonly employed in combination with other illegal drugs, particularly MDMA [4], and is considered to be favorite on the global drug market [5]. In addition, 2C-B is among the chemical compounds most often found as an adulterant when analyzing MDMA tablets [6, 7] which signifies that many individuals have consumed it unknowingly.

2C-B is classified as a Schedule II Drug under the Convention on Psychotropic Substances [8] and has a growing presence on the illegal market; nevertheless, the scientific literature concerning its properties is scarce. There are a number of preclinical studies that demonstrate its action. 2C-B has been reported to increase dopamine and decrease dihydroxyphenylacetic acid concentrations in the nucleus accumbens. It has a biphasic action on locomotion, showing inhibition at low doses and excitatory effects at higher ones [9]. In a similar manner to MDMA, it is an inhibitor of the serotonin transporter and, to a lesser extent, acts on the norepinephrine and dopamine transporters [10–12]. Whilst 2C-B modifies visual perception, it has been reported to have limited efficacy as a 5-HT-2A receptor partial agonist, the usual mechanism of hallucinogenic phenylethylamines [13–15]. The other research, however, has suggested that it acts as a 5-HT-2A receptor antagonist [15] and a partial agonist of 5-HT-2C receptors [11, 15]. It is of interest that some other 2C-derivatives, such as 2C-B-FLY and 25B-NBOMe, have been recently introduced on the drug market and present strong hallucinogenic properties. Like MDMA and 2C-B, 2C-B-FLY and related benzofuran compounds inhibit noradrenaline and serotonin uptake more than dopamine uptake; in addition, they are partial 5-HT-2A and 5-HT-2b receptor agonists. These compounds have intense hallucinogenic properties and present a risk for vasoconstriction, and some fatalities have been reported [12]. 25B-NBOMe is a very potent 5-HT-2A receptor agonist, with high 5-HT-2A/5-HT1A selectivity, and shows affinity for adrenergic alpha-1 receptors. It produces intense hallucinogenic effects at very low doses (100–200 micrograms) and can induce severe intoxication and fatalities [16]. 2C-B is metabolized by the monoamine oxidases A and B and the cytochrome P450 [17, 18].

Prevalence of 2C-B consumption in a recent sample of Australian NPS users was 8% [3] whilst in a sample of 230 Spanish research chemicals users it was the most frequent drug employed during their lifetime. A number of participants obtained the substance from the internet. It was usually taken at home, followed by open-air settings and, to a lesser extent, in closed spaces such as clubs. The main reasons for its use were experimental or psychonautic, recreational, spiritual, and, in some subjects, therapeutic [4]. In general, the substance is marketed as tablets and taken by the oral route. An average dose is considered to be 20 mg with 8–10 mg producing mild effects [4, 19]. The patterns of use are similar to other NPS including 2C-derivatives [16, 20, 21].

Studies concerning its subjective effects [4, 19] suggest a profile similar to ayahuasca and* Salvia divinorum* with respect to perception and comparable to MDMA regarding emotional, cognitive, and volitional components. In addition, 2C-B appears to have a higher rating than amphetamine on the scales of pleasure, sociability, and sedation and a lower one than MDMA with respect to psychosomatic anxiety. The possibility of 2C-B to stimulate sensuality and sexuality has been suggested [22]. To date, no fatalities due to overdose have been attributed, and only one case of psychosis [23] and one of cerebral vasculopathy have been described [24], both of which developed 48 hours after ingestion. Nevertheless, in neither of the cases was the presence and/or exclusivity of 2C-B confirmed in the biological samples. In spite of the low rate of accidents related to this substance there exist data advising of intersubject variations regarding susceptibility to 2C-B toxicity [25]. Descriptions of severe medical and psychiatric reactions after the use of substances with similar profiles suggest precaution in its use [16, 26].

In general, the development of controlled clinical studies with illegal psychoactive drugs is hindered by legal considerations including the problems of lawfully obtaining the drugs. For some time, however, there has been a resurgence of interest in the possible therapeutic uses of illegal substances [27–30]. An example of this is MDMA which has been studied in the treatment of posttraumatic stress disorder [31–34]. It appears that the pharmacological action of MDMA deactivates the amygdala during the reliving of traumatic experiences and, through the repetition of this action the emotional activation of these memories can be extinguished [35]. Given its similarities with MDMA it has been suggested that 2C-B could be used in a similar manner to resolve emotional conflict [36, 37].

Present knowledge regarding the pharmacology of 2C-B is insufficient to clearly permit its definitive categorization as a stimulant, hallucinogen, or entactogen (i.e., “to produce a touching within” and introspection) [38], although there are references to its empathogenic quality (i.e., the ability to feel and share another person's emotions) in the literature [15]. The principal objective of this study was to explore the emotional effects of 2C-B in order to establish its correct classification.

## 2. Materials and Methods

### 2.1. Participants

The protocol was approved by the Local Research Ethics Committee (CEIC-Parc de Salut Mar, Barcelona, Spain) and the study conducted in accordance with the Declaration of Helsinki. Volunteers were recruited through the Association for the Study of States of Consciousness (PHI). They signed an informed consent and were financially compensated for their participation.

Inclusion criteria were acknowledged previous use of 2C-B and did not consume any type of psychoactive substance during the 48 hours prior to the study. Exclusion criteria were a history of any serious medical or psychopathological condition including history of drug dependence (except for tobacco) or previous psychiatric comorbidities, previous adverse reactions to 2C-B, and taking chronic medication.

### 2.2. Procedure

The sessions took place at the home of a member of the PHI Association. Baseline evaluation was performed from 10.00 to 12.00 after which the participants had light breakfast; two hours later (approximately 14.00) 2C-B was self-administered. A dosage of 20 mg was employed as the same amount had been previously consumed by the participants without any adverse effects. Gas chromatography analysis was carried out by Energy Control, a harm reduction nongovernmental organization (http://www.energycontrol.org/), and showed the 2C-B to have 95% purity. Urine samples were collected for drug testing before the session (amphetamines, barbiturates, benzodiazepines, cocaine, methamphetamine, morphine, methadone, phencyclidine, marijuana, and MDMA) (Instant-View, Multipanel 10 Test Drug Screen Alfa Scientific Designs Inc., Poway, CA, USA).

Measurements were carried out at baseline (prior to 2C-B self-administration) between 1.5–3 h (peak effects) and 6 h after dose. During this time range the following were performed (always in the same order): measurement of vital signs; saliva collection; International Affective Picture System (IAPS) test; Face Emotion Recognition Task (FERT); speech evaluation; and subjective effects questionnaires (POMS, visual analog scales, ARCI, VESSPA, and HRS). In order to avoid adaptation in the IAPS and FERT, two distinct sets, calibrated according to the normative data and counterbalanced for each condition, were constructed. This was also applied in the FERT changing the established order of slide presentation.

### 2.3. Neuropsychological Testing

The following neuropsychological tests were performed at baseline and at peak effects (1.5 h).

The IAPS consists of a set of standardized emotional stimuli. It was designed by Lang et al. [39] for experimental research into emotions and attention. For the purposes of this study 120 stimuli were classified into two different sets (60 in each) divided equally into the following categories: positive, negative, and neutral. The creation of both sets was based on standards from the Spanish version published by Moltó et al. [40] and Vila et al. [41]. Each stimulus was applied for 2 seconds and an evaluation allocated from 0 to 9 for “valence” and “arousal.” The former indicated the degree of pleasure, happiness, and well-being that the participants experienced when they observed the image; the latter referred to the arousal or excitement it produced.

The FERT is created by Ekman and Friesen [42]. Six basic emotions are portrayed in a series of monochromatic photographs (ten per emotion). The photographs are shown to the participants; during each series the expression of the emotion is gradually intensified by 10% from 0% (neutral) to 100% (standard). For this study four actors were employed (2 men and 2 women) to express the following emotions: happiness, sadness, anger, disgust, and fear. Each image was presented for the duration of one second.

Finally, a task to evaluate free speech was performed. The volunteers were asked to talk for three minutes about a specific theme (family or friends) which was counterbalanced for each study phase (baseline and peak effects). Prior to carrying out the task the participants were required to reflect for five minutes on their theme. They spoke individually in separate rooms in the presence of the first author (Débora González) who, employing nonverbal communication, actively listened without interfering at any time in the monologue. The speeches were recorded with a Zoom H4n device. Later, a group of five judges specialized in psychology and blinded to the experimental conditions evaluated the following dimensions on a scale from 0 to 7: “coherence” (understood as congruence, possessing a logical structure without contradictions); “emotionality” (referring to the emotions that the speech awakens in third parties); “depth” (regarding how the speaker delves into the theme as opposed to superficiality); and “focus” (that is to say, the content of the speech is centered on the theme and not dispersed). Similar categories have been employed to evaluate speech under the effects of MDMA and amphetamine [43]. Verbal fluency was calculated as an additional variable by measuring the number of syllables employed in free and spontaneous speech without taking into account the forced speech produced to finish the three minutes of the task. Explicit declarations and questions such as “I don't know what to say anymore” and “How much time is left?” plus pauses of longer than 5 seconds were considered to be examples of forced speech. The aim of this task was to establish whether 2C-B could modify verbal behavior in such a way that it could be appreciated by a professional within the context of clinical psychotherapy.

### 2.4. Questionnaires regarding Subjective Effects

A large number of visual analogue scales (VAS) and questionnaires (ARCI and VESSPA) to evaluate subjective effects were completed at base line, peak effects, and 6 h (summary of the 0–6 h effects). The POMS questionnaire was applied at baseline and peak effects, and the HRS questionnaire was completed only at 6 h after dose (summary of the 0–6 h effects).

VAS (100 millimeters (mm)) labeled with different adjectives marked at opposite ends with “not at all” and “extremely” were used [44, 45]. Subjects were asked to rate effects of intensity, high, good effects, bad effects, liking, changes in distances, changes in colors, changes in shapes, changes in lights, hallucinations-seeing of lights or spots, hallucinations-seeing animals, things, insects, or people, changes in hearing, hallucinations-hearing sounds or voices, drowsiness, dizziness, confusion, fear, depression, or sadness, different or changed unreal body feeling, unreal body feeling, different surroundings, and unreal surroundings.

The Addiction Research Center Inventory (ARCI) was developed by Haertzen et al. [46] and the Spanish version later validated by Lamas et al. [47]. It consists of five scales: (i) “morphine-benzedrine group” (MBG), measuring euphoria; (ii) “pentobarbital-chlorpromazine-alcohol” group (PCAG), measuring sedation; (iii) “lysergic acid diethylamide” scale (LSD), measuring somatic-dysphoric effects; (iv) “benzedrine” group (BG), a stimulant scale consisting mainly of items relating to intellectual efficiency and energy; and (v) “amphetamine” (A), an empirically derived scale sensitive to the effects of d-amphetamine.

The Evaluation of Subjective Effects of Substances with Abuse Potential questionnaire (VESSPA-SEE) was originally created in Spanish by Poudevida et al. [48] to measure the effects of MDMA; it was later used for the evaluation of other substances [49]. It is composed of 36 Likert items grouped into 6 scales: sedation, psychosomatic anxiety, changes in perception, pleasure and sociability, activity and energy, and psychotic symptoms.

The POMS questionnaire was designed by McNair et al. [50] to accurately evaluate possible alterations in mood states. Its validated Spanish version [51] is made up of 48 Likert items which range from 0 (“no” to everything) to 4 (“extremely”) distributed in 6 scales: anger, depression, tension, tiredness, vigor, and friendship.

The Hallucinogenic Rating Scale (HRS) was first created by Strassman et al. in 1994 [52] and a validated Spanish version was performed by Riba et al. in 2001 [53]. It consists of 71 Likert items distributed in 6 scales: (i) “somaesthesia” (reflecting somatic effects); (ii) “affect” (sensitive to emotional and affective responses); (iii) “volition” (indicating the person's degree of impairment); (iv) “cognition” (describing changes in thought process or content); (v) “perception” (measuring visual, olfactory, gustatory, and auditory experiences); and (vi) “intensity” (reflecting the strength of the overall experience).

### 2.5. Vital Signs and Saliva Samples

Vital signs (heart rate and blood pressure) were measured with an automatic Omron monitor at baseline, peak effects (1.5 h), and 6 h after dose. Saliva samples were collected with a Salivette device at baseline (prior to self-administration), 1.5 h after administration (peak effects), and 6 h after administration. Concentrations of 2C-B in saliva were analyzed by gas chromatography coupled to mass spectrometry (data not shown).

### 2.6. Statistical Analysis

The neuropsychological test scores were contrasted with paired Student's *t*-test (baseline versus peak effects). The results of the subjective effects scales were compared with paired Student's *t*-test (baseline versus peak effects and baseline versus 6 h, depending on the questionnaire). In the case of vital signs a one-way analysis of variance-ANOVA with repeated measures (baseline, peak effect (1.5 h), and 6 h) and a Tukey test post hoc comparison between times were performed. In order to observe intergender differences, an independent sample *t*-test was carried out with baseline and postdose measures for neuropsychological tests and POMS. Analyses were performed with the SPSS 12.0 program. *P* value < 0.05 was considered statistically significant.

## 3. Results

### 3.1. Subjects

The sample was composed of 20 participants (12 women) with a mean age of 34.65 years (SD = 5.25; range 27–49 years) and a body mass index of 22.14 (SD = 2.56; range 18–24). 60% of the volunteers had a university degree and the mean previous 2C-B recreational consumption was 6.5 times (SD = 7.0; range 2–30). All the volunteers had previous recreational experience with amphetamines, MDMA, hallucinogens, cocaine, and cannabis, 16 were currently smokers (from 2 to 20 cigarettes/day), and their mean daily alcohol consumption was 10 g. All baseline drug urine tests were negative. The participants did not show any signs of intoxication at the baseline evaluation.

### 3.2. Neuropsychological Testing

In the IAPS task (Table 1), 2C-B produced a significant reduction in the valence of the negative stimuli (*P* = 0.037) which implies that under the effects of 2C-B there is a tendency to evaluate negative stimuli as being the most disagreeable and the cause of the greatest discomfort. Even though the valence of the positive and neutral stimuli tended to augment in the experimental setting, activation remained practically the same between both conditions with the exception of the negative stimuli which had a tendency to increase under 2C-B effects (*P* = 0.11). There were no differences between genders in the evaluation of emotional stimuli.

In the FERT (Table 1) a significant increase was observed after 2C-B in the rate of errors in the recognition of expressions of happiness (*P* = 0.027). However, although happiness was the emotion best detected at baseline, under the effects of 2C-B, fear predominated. Intergender analysis revealed that the larger number of errors in detecting expressions of anger was observed in the male participants (men = 5.75 (SD = 2.12) versus women = 3.75 (SD = 1.06); *P* = 0.035).

The number of syllables produced in the participants' free speech after 2C-B did not vary along the study (474.74 (SD = 216.23) versus 479.11 (SD = 152.089); *P* = 0.936). In spite of a considerable intersubject variability, differences were not observed between genders. Speech was observed to be more emotional under the effects of 2C-B (*P* = 0.012) (Table 1), intergender analysis revealing a higher rating of this variable in the female participants (men = 4.36 (SD = 0.84) versus women = 5.57 (SD = 1.12); *P* = 0.042). Given the repercussion concerning the influence of 2C-B on emotion and speech, it should be noted that two of the female participants cried whilst performing this task and one male participant had an attack of spontaneous laughter which made his speech unintelligible for the judges. As a result, he was excluded from the final sample (*n* = 19).

### 3.3. Questionnaires regarding Subjective Effects

Administration of 2C-Bproduced significant increases in all of the VAS at severe effects and/or 6 h (summary) for positive effects, changes in perceptions, and mild hallucinogenic effects (mainly visual). No differences were observed between genders.

In the ARCI questionnaire 2C-B induced significantly high scores for euphoria (MBG) and dysphoria-somatic effects (LSD) and for the two scales related to amphetamine effects (BG and A). No changes were reported in sedation (PCAG). Differences were not observed between genders.

Significant differences were found for the VESSPA questionnaire in all of the subscales. The highest scores were reported in pleasure and sociability and the lowest ones in psychosomatic anxiety and psychotic symptoms. The intersubject analyses showed differences between genders with respect to the scale of activity and energy at 6 h after dose (men = 9.88; (SD = 4.91); women = 5.00; (SD = 4.51); *P* = 0.041).

In the POMS questionnaire a significant reduction in anger under the effects of 2C-B was reported in spite of the low baseline levels (*P* = 0.042). Differences were not found, however, in the scales of vigor, depression, tiredness, tension, and friendship for any of the groups (Table 1). The intergender analysis showed a significant reduction in men, but not in women, of tiredness under the effects of 2C-B (men = 0.50; (SD = 0.535) versus women = 3.75 (SD = 3.19); *P* = 0.011).

Finally, the HRS questionnaire had high scores for most of the scales at 6 h after dose, particularly in volition, intensity, and perception. The lowest score was for cognition.

### 3.4. Vital Signs and Saliva

Discreetly statistical significant increases, in comparison to baseline, in heart rate (+6 beats/min), systolic (+5 mmHg) blood pressure and diastolic (+4 mmHg) blood pressure were observed at 1.5 h, and baseline values were recovered at 6 h. No differences were reported between genders. Concentrations of 2C-B were found in saliva at 1.5 and 6 hours (6.8 ng/mL and 2.3 ng/mL, resp.) (data not shown).

## 4. Discussion

This study is the first to describe the emotional effects of 2C-B and one of the few that reports subjective and cardiovascular effects after its administration [19].

Our results indicate that 2C-B has specific effects on emotional processing and mood states which permit its classification as an entactogenic substance with psychedelic and hallucinogenic characteristics. Entactogenic effects are characterized as an “open mind” state with properties including an increase in self-awareness, the sensation “to produce a touching within,” introspection, elevated sensory perception, and enhanced prosocial effect [54]. However, its influence with respect to the worsening of the affective perception of faces does not point to empathogenic qualities and, despite the slight effects observed in cardiovascular measurements, the result of the verbal fluency test is not typical of stimulants.

Serotonin is an ancestral neuromodulator that modulates the control of violent impulses incited by provocation through a down-top regulation of emotional reactions [49, 55]. In this regard, the effects of 2C-B are characteristic of the activation of serotonergic receptors as a significant decrease of anger was observed in the POMS questionnaire. The increase in well-being in the VAS and sociability in the SOC subscale of the VESSPA reaffirms this result, given that the subscale was created to evaluate the typical effects of entactogenic substances, reflecting the pleasurable effect of feeling well with oneself and others [48]. Such effects are similar to those induced by MDMA [44, 45] and 2C-B [19] as previously reported. Nevertheless, the effect of 2C-B on mood states reflected by the POMS differs substantially from that of MDMA and amphetamine, in spite of sharing a similar chemical structure, as these two substances tend to augment vigor and friendship [49–60]. Oxytocin, liberated by MDMA, has been more strongly associated with the latter sentiment than serotonin [61, 62]. While the specific role of oxytocin with regard to the subjective effects of MDMA remains unclear, it is possible that this hormone does not have a strong influence on 2C-B effects.

Contrary to our expectations, under the effects of 2C-B, emotional reactivity with respect to the negative stimuli in the IAPS task increased. This type of affective response is uncommon and has not been observed in other substances. It is well-known that with any kind of stimulus amphetamine augments valence and arousal [63]. In the Multifaceted Empathy Test (MET), MDMA increased implicit empathy (arousal) particularly with positive valence stimuli [64]. On the other hand, high doses of alcohol suppress the response to negative stimuli [65]. In all of these cases there is some form of affective regulation that has been linked to heightened response as they either strengthen the impact of the positive stimuli or reduce the negative ones [63]. In contrast, 2C-B does not appear to produce this type of response as greater discomfort with respect to negative stimuli at baseline was reported.

It is noteworthy that, under the effects of 2C-B, affective perception deteriorated and the number of errors in recognizing positive expressions increased. A similar pattern has been observed in depressed patients [66] which reverses with prolonged administration of SSRI antidepressants [67, 68]. Most studies, however, which examined the effect of SSRI on the affective processing of faces have found that an acute administration of a single dose helped recognition of expressions of fear [69, 70] with a corresponding worsening at long term administration [71–73].

In our study, in spite of not having observed significant differences in the recognition of expressions of fear, it did, nevertheless, become the emotion best detected under the effects of 2C-B. Some authors have reported an increase in the accuracy of detecting fear under the influence of MDMA followed by a significant decrease 4 days after ingestion [74]. Most studies, however, have concluded that MDMA worsens recognition of negative emotional expressions [57, 75, 76] particularly those of fear [57]. Such results can be extrapolated to other substances that act upon the 5-HT-2A such as psilocybin, the effect being reversed when these receptors are blocked with ketanserin [77]. A recent study has observed the role of the 5HT-1A receptors in the recognition of fear when the agonist buspirone was administered [78]. It was found that the 5HT-1A and 5-HT-2A receptors are linked to the structural coding of this expression in a prior process, both receptors thus contributing to its reduced recognition. Notwithstanding the fact that 2C-B alters visual perception, our results underscore the issue that its unique profile in the affective processing of faces leads to further questions regarding the possible implications of other receptors, such as 5HT-2C, in this kind of process.

In a similar manner to MDMA and in contrast to methamphetamine [37] and* d*-amphetamine [79], verbal fluency did not increase under the effects of 2C-B: significant differences were not observed in the number of syllables uttered during the experiment. Differences were, however, reported in the emotionality and depth variables, particularly in the female participants. A recent study has demonstrated that under the effects of 1.5 mg/kg of MDMA discourse comes close to concepts such as “friend” and “support” and with 0.75 mg/kg to “empathy.” However, speech under the effects of 20 mg of methamphetamine distances itself from concepts such as “compassion” [80]. Considering that two of the female participants cried whilst performing the free speech task, it appears that 2C-B influences emotionality, reflecting an entactogenic activity defined by Nichols [81] as “to produce a touching within.”

The intergender variable should be taken into account with respect to 2C-B effects. In the POMS questionnaire a reduction in tiredness was observed in men, but not in women. In addition, in the speech task the female participants had significantly higher scores in emotionality. These results show that 2C-B has similar effects to amphetamine in men and more entactogenic effects in women. A similar condition occurs with amphetamine, the effects of which depend not only on gender but also on the menstrual phase. Women barely experience stimulation during the luteal phase due to an increase in the plasmatic levels of progesterone [82]. This fact draws attention to one of the limitations of the study as the menstrual phases of the female participants were not taken into consideration. Given the relevant influence of hormones in subjective emotional states such data should be included in future research.

The 2C-B dose that was administered provoked minor alterations in the participant's vital signs, with a sympathomimetic stimulation similar to an average dose of MDMA [83]. There are no available data regarding 2C-B concentrations in plasma and saliva. As occurs with weak bases, such as MDMA and methylphenidate, the saliva concentrations could be related to the plasma ones [84, 85].

Our study has some limitations related to its before-after design and lack of control (placebo or MDMA). In addition, subjects were assessed in a nonexperimental setting and the number of evaluations was limited. The use of the same dose for all subjects was a strength although few differences were observed with respect to gender. Whilst the standard rapid urine drug test employed prior to the session was negative for the most common drugs, it was naturally unable to identify any other possible NPS (e.g., synthetic cannabinoids or cathinones). The participants did not, however, present any signs of intoxication. With respect to tolerability and safety, all the volunteers had had previous positive experiences with 2C-B and other drugs. Nevertheless, taking into account the fact that substances with similar profiles can induce severe medical and psychiatric reactions, its consumption in naïve users or in subjects with less experience could produce side effects.

## 5. Conclusions

In spite of its limitations our study demonstrates that a single dose of 2C-B influences the bottom-up and top-down processes of emotional regulation and has an effect on emotional state, affective processing, and behavior. The increase of reactivity to negative stimuli, the enabling of emotional expression through speech, and heightened feelings of well-being with oneself and others reflect the entactogenic activity of the substance. The sum of these effects on emotional regulation, together with the decrease of anger observed in the POMS, could make 2C-B a potentially useful psychotherapeutic tool to treat changes of attitude in disorders related to violence and aggression through affective reevaluation. At present there is heated debate regarding the therapeutic potential of psychedelic drugs and their legal reclassification in order to investigate their potential use as medication [86, 87]. In a similar fashion to other NPS, 2C-B is widely available online to anyone (including adolescents and vulnerable individuals), and the risks of falsification and adulterants must be considered. Due to the fact that the recreational use of substances with similar profiles can induce severe medical and psychiatric reactions, precaution must be taken with respect to its use. As occurs with all NPS, data is not currently available concerning the medium-long term health consequences of 2C-B use [88]. Additional research regarding its pharmacology and toxicity is required in order to assess its possible therapeutic effects and potential health risks.

## Figures and Tables

**Table 1:** Effects of 2C-B in neuropsychological test (IAPS, FERT, and interjudge scores for speech) and the Profile of Mood States (POMS) questionnaire at baseline and after 2C-B administration (+1.5 h, peak effects).

	Baseline		Peak effects		*P* value
	Mean	SD	Mean	SD	
IAPS					
IAPS valence					
Positive	7.31	0.70	7.42	0.63	0.391
Neutral	5.18	0.34	5.30	0.37	0.209
Negative	3.22	0.60	2.97	0.60	**0.037**
IAPS activation					
Positive	4.88	1.14	4.86	0.99	0.909
Neutral	5.11	0.38	5.09	0.55	0.789
Negative	6.91	0.53	7.13	0.66	0.110

FERT					
Happiness	1.90	1.17	2.90	1.65	**0.027**
Sadness	4.50	1.43	4.90	1.71	0.226
Anger	4.55	1.54	4.40	1.85	0.904
Disgust	5.20	1.64	4.80	1.32	0.442
Fear	3.25	1.25	2.70	1.13	0.102

Interjudge scores for speech					
Coherence	5.13	0.996	4.57	0.935	0.101
Emotionality	4.13	0.952	4.98	1.074	**0.012**
Depth	4.21	1.239	4.74	0.924	0.071
Focus	4.84	1.152	4.23	1.023	0.116

POMS					
Vigor	9.30	2.47	9.65	5.09	0.774
Depression	2.65	2.82	1.45	2.69	0.065
Friendship	15.90	2.22	14.85	3.17	0.138
Tiredness	4.60	3.52	2.45	2.95	0.074
Anger	1.95	3.30	0.30	0.73	**0.042**
Tension	2.80	5.94	3.05	4.77	0.826
